# Malignancy yield and clinical predictors in radiologically detected adult urachal anomalies: A retrospective cohort study

**DOI:** 10.1002/bco2.70284

**Published:** 2026-10-06

**Authors:** Per Blomqvist, Lala Joulha Ian, Emelie Holmin, Suleiman Abuhasanein

**Affiliations:** ^1^ Department of Urology, Institute of Clinical Science Sahlgrenska Academy, University of Gothenburg Gothenburg Sweden; ^2^ Department of Surgery, Urology Section NU‐Hospital Group Uddevalla Region Västra Götaland Sweden; ^3^ Bra Liv Hälsan. Primary Care Centre Region Jönköping County Sweden; ^4^ Department of Research and Development NU‐Hospital Group Trollhättan Region Västra Götaland Sweden

**Keywords:** adult, asymptomatic, bladder cancer, haematuria, incidental findings, malignancy risk, urachal anomalies, urachus

## Abstract

**Objective:**

To evaluate the occurrence of bladder malignancy or papillary urothelial neoplasm of low malignant potential (PUNLMP) detected at initial evaluation or during follow‐up among adults with radiologically reported urachal anomalies, and to explore clinical factors associated with neoplasia.

**Materials and methods:**

We conducted a retrospective single‐centre cohort study. Adult patients with radiologically reported urachal anomalies between 2000 and 2024 were identified through institutional radiology information system and report archive and underwent detailed chart review. Patients with a prior history of bladder or upper urinary tract malignancy were excluded. Clinical presentation, imaging findings, cystoscopy, histopathology and outcomes were analysed. Clinical variables associated with malignancy were explored using Firth penalized logistic regression.

**Results:**

A total of 245 patients were included: 92/245 (38%) had incidental/asymptomatic findings and 153/245 (62%) were symptomatic. Bladder malignancy/PUNLMP was detected in 14/245 patients (5.7%) overall, including 0/92 incidental/asymptomatic patients (0%) and 14/153 symptomatic patients (9.2%). Macroscopic haematuria was present in 12/14 patients with malignancy/PUNLMP and was associated with a higher frequency of neoplasia: 12/66 patients with haematuria (18%) versus 2/179 without haematuria (1%; p<0.001). In exploratory multivariable analysis, macroscopic haematuria remained associated with malignancy/PUNLMP (OR 5.29, 95% CI 1.10–39.5). Most detected tumours were of non‐urachal histology, and 8/14 (57%) were stage T2 or higher.

**Conclusions:**

Incidentally detected urachal anomalies in adults were not associated with malignancy during follow‐up, suggesting that conservative management may be appropriate in asymptomatic patients. In contrast, symptomatic presentation, particularly macroscopic haematuria, warrants thorough urological evaluation due to its association with a substantially increased risk of bladder malignancy.

## 1 INTRODUCTION

The urachus, a tubular embryological structure located in the extraperitoneal space of the Retzius, originates from degeneration of the allantois and cloaca and extends between the dome of the urinary bladder and the umbilicus. It obliterates in the late embryonic period and subsequently forms the median umbilical ligament^1^, ^2^, ^3^, ^4^, ^5^ (Figure 1). Urachal anomalies are uncommon, with historical estimates around 1 in 5000 births and lower persistence/detection in adults, and are more common in males (67%).^4^, ^6^ Incomplete obliteration of the urachal tract may result in one or more of four recognized anomaly types: urachal cyst, urachal sinus, patent urachus and vesicourachal diverticulum.^3^, ^4^, ^6^

**FIGURE 1:**
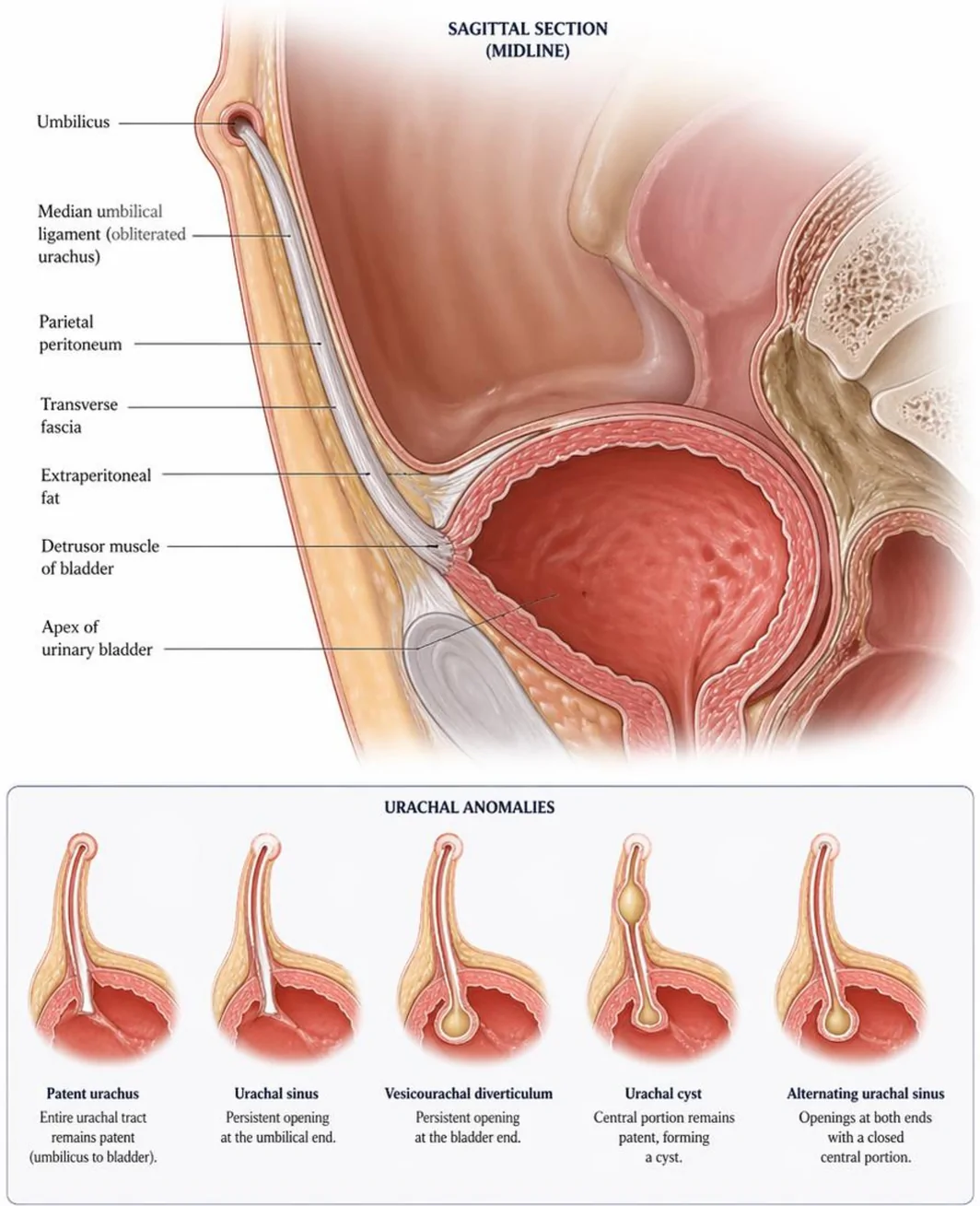
Urachus: embryological development, anatomy and clinical significance. Figure generated with assistance from ChatGPT‐Sora (OpenAI).

The presentation of urachal anomalies is heterogeneous, ranging from asymptomatic incidental findings to symptomatic disease. With increased use of medical imaging, incidental detection of urachal anomalies during investigations unrelated to urinary tract pathology has become more common. Symptomatic patients may present with umbilical drainage, abdominal masses, haematuria, urinary tract infections (UTI) and mucinuria. Late complications of urachal anomalies include calculi/stone formation, recurrent UTIs and malignant transformation.^3^, ^7^, ^8^

Bladder adenocarcinoma is rare, accounting for approximately 0.5%–2% of all bladder malignancies.^9^ Urachal adenocarcinoma represents a distinct and even rarer subtype with specific diagnostic criteria and biological behaviour. Diagnostic criteria for urachal carcinoma have historically been based on anatomical and pathological definitions first formalized by Sheldon et al.,^10^ requiring exclusion of primary bladder origin and confirmation of midline dome location subtype. Although pathogenesis remains incompletely understood, chronic inflammation, urinary stasis and subsequent metaplastic or dysplastic changes have been proposed as mechanisms contributing to malignant transformation.^11^, ^12^

Consequently, the optimal management of urachal anomalies—particularly those detected incidentally in adults—remains controversial, with no clear consensus.^2^, ^6^ While some authors advocate routine prophylactic excision to mitigate potential malignant risk,^1^, ^3^, ^13^ others support a more conservative approach given the presumed low incidence of malignancy.^6^, ^11^ Therefore, this study aimed to estimate the risk of histologically confirmed bladder malignancy or papillary urothelial neoplasm of low malignant potential (PUNLMP) following radiological detection of urachal anomalies in adults, with a particular emphasis on incidental/asymptomatic findings and on whether identified malignancies were likely to be of urachal origin.

## 2 MATERIALS AND METHODS

### 2.1 Study design and data collection

This retrospective cohort study was conducted at a single tertiary care centre. Patients were identified through a free‐text search of all finalized radiology reports stored in the institutional radiology information system and report archive between 1 January 2000 and 31 December 2024. The search included reports from (ultrasonography, non‐contrast CT, contrast enhanced CT, CT urography, MRI) using the English/Latin search terms ‘urachus’ and ‘urachal’ together with the swedish terms ‘urakus’ and ‘urakal’. Individual electronic medical records were subsequently reviewed to verify the presence of urachal anomalies.

Patients were eligible if they were ≥18 years of age at the index examination and had a radiologically reported urachal lesion. Reports containing negated terms or mentioning the urachus solely as an anatomical landmark or unconfirmed differential diagnosis were excluded prior to extended review. Patients with a previous history of urinary bladder cancer (UBC) or upper urinary tract malignancy (UTUC) were also excluded.

Clinical presentation was classified according to symptoms and indications documented at the time of the index radiological examination. Patients were categorized as symptomatic if the imaging investigation was prompted by macroscopic haematuria, lower urinary tract symptoms, urinary tract infection, umbilical discharge or infection, umbilical mass or clinically relevant abdominal pain. Patients were classified as incidental/asymptomatic when the urachal anomaly was identified on imaging performed for unrelated reasons and no urachus‐related symptoms were documented.

Radiological reports were reviewed and urachal anomalies were categorized as patent urachus, urachal cyst, urachal sinus, vesicourachal diverticulum, focal bladder wall thickening suspected to involve the urachus or unspecified urachal anomaly. Classification was based on the terminology used in the original radiology reports. Lesions lacking sufficiently detailed description for subtype classification were categorized as unspecified urachal anomalies. No systematic central re‐review of the original imaging studies was performed; the classifications thus reflect real‐world radiological reporting rather than standardized retrospective image adjudication.

Data were extracted retrospectively from institutional electronic medical records. Collected variables included demographic characteristics, imaging modality, radiological findings, presenting symptoms, cystoscopic findings, surgical interventions, histopathological results, tumour stage and grade, survival status and dates of diagnosis and follow‐up. Cystoscopy was not mandated by a study protocol but was performed according to clinical judgement, based on presenting symptoms, imaging findings and urological assessment. Consequently, diagnostic work‐up differed between symptomatic and incidental/asymptomatic patients. Contrast enhancement was assessed only when contrast‐enhanced cross‐sectional imaging was available. Enhancement was recorded as present or absent. Patients evaluated only by non‐contrast imaging were classified as not assessable and were not considered to have absent enhancement.

### 2.2 Statistical analysis

The primary outcome was histologically confirmed malignancy considered to arise from, or directly involve, the urachal remnant, based on tumour location, imaging findings, operative notes, cystoscopic findings and histopathological reports. The secondary outcome was any histologically confirmed bladder malignancy or PUNLMP detected at index evaluation or during follow‐up.

Continuous variables were expected to show skewed distributions because of potential outliers and small subgroup sizes and were therefore summarized using medians and interquartile ranges (IQR). Categorical variables were presented as frequencies and percentages. Fisher's exact test was used for two‐group comparisons of categorical variables because of small sample sizes and low expected cell counts. Comparisons involving larger contingency tables were analysed using the chi‐square test when assumptions for this method were met. Continuous variables were compared using the Mann–Whitney *U* test.

Associations with bladder malignancy/PUNLMP and surgical intervention were explored using Firth penalized logistic regression, which reduces small‐sample bias and mitigates separation in sparse datasets.^14^, ^15^ Given the limited number of outcome events, all regression analyses were considered exploratory. Variables were selected a priori based on clinical relevance and data availability. To reduce overfitting, findings were interpreted primarily according to effect sizes, confidence interval widths and clinical plausibility rather than *p*‐values alone. All statistical tests were two‐sided, and *p*‐values <0.05 were considered statistically significant. Descriptive analyses were conducted using SPSS Statistics version 30 (IBM Corp., Armonk, NY, USA), whereas Firth penalized logistic regression analyses were conducted using R version 4.5.2 (R Foundation for Statistical Computing, Vienna, Austria).

## 3 RESULTS

All potentially eligible patients identified through the initial free‐text search (*n* = 351) were assessed for study inclusion. Reports not fulfilling predefined inclusion criteria due to age below 18 years at assessment (*n* = 27), absence of documented urachal remnants (*n* = 17), and duplicate registrations (*n* = 12) were excluded at this initial stage. The remaining patients underwent full eligibility review during which a further 50 were excluded due to unconfirmed urachal pathology (*n* = 34) and previous urological malignancy (*n* = 16) (Figure 2).

**FIGURE 2:**
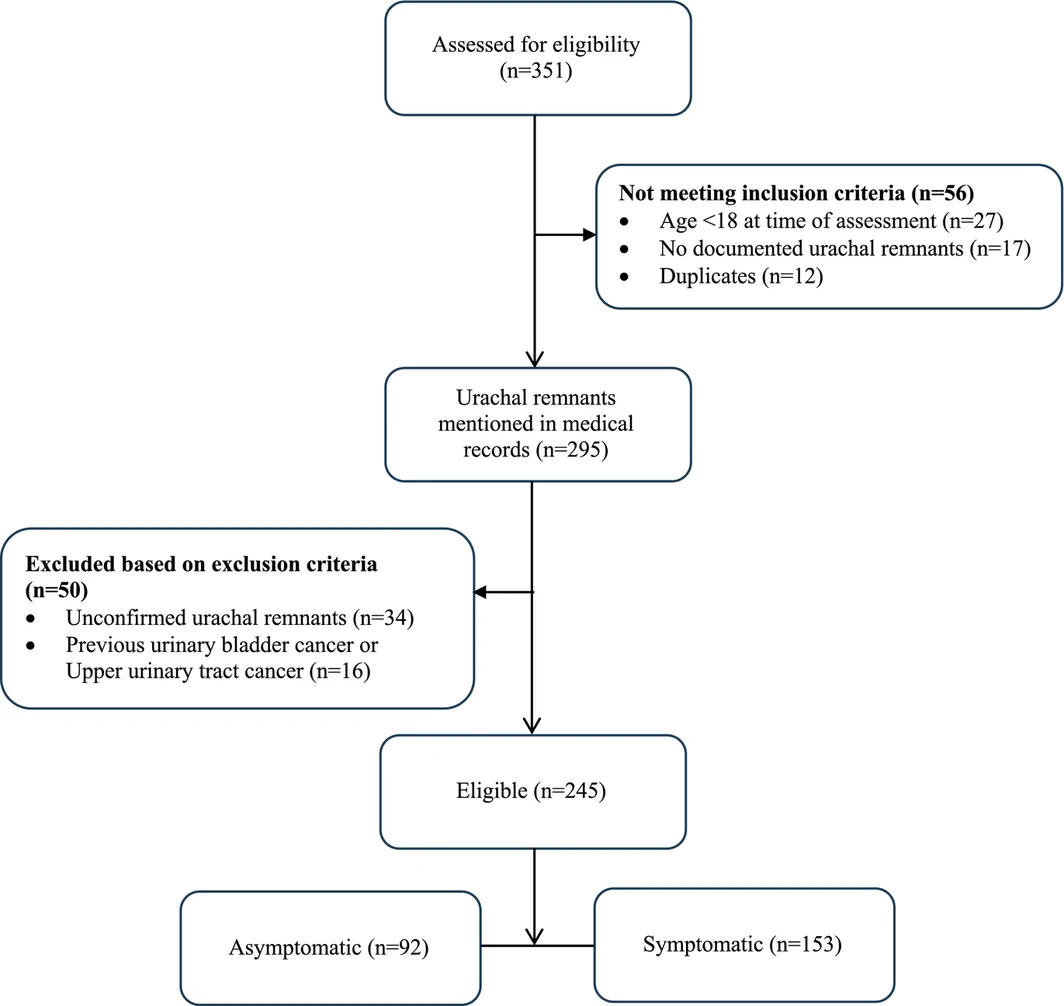
Flow diagram of study selection and eligibility assessment.

Ultimately, 245 adult patients with radiologically reported urachal anomalies were included in the study. Of these, 92 patients (38%) had incidentally detected or asymptomatic findings, whereas 153 patients (62%) presented with symptoms at diagnosis (Figure 2). No malignancies were identified among patients with incidental or asymptomatic urachal anomalies. In contrast, 14 cases of malignancy or PUNLMP were detected in the symptomatic group.

The median age was similar between symptomatic patients and those with incidental findings (60 vs. 64 years, *p* = 0.154). Female sex was more common in the symptomatic group (37% vs. 22%, *p* = 0.016). Median follow‐up duration was 9 years in both groups (*p* = 0.211), and mortality during follow‐up did not differ significantly (20% vs. 28%, *p* = 0.162). Smoking status was comparable between groups (*p* = 0.406) (Table 1).

**TABLE 1:** Descriptive parameters of all patients included in the study.

Variable		Symptomatic	Incidentaloma	Overall	*p* value
Nr	(%) of the line	153 (62)	92 (38)	245	
Age (years)	Median, (IQR)	60 (34–75)	64 (49–75)	62 (41–75)	0.154
Gender	Female	56 (37)	20 (22)	76 (31)	0.016
Follow‐up (years)	Median, (IQR)	9 (6–13)	9 (6–11)	9 (6–12)	0.211
Death during follow‐up	Yes	31 (20)	26 (28)	57 (23)	0.162
Smoking status^a^	Never smoked	76 (56)	45 (55)	121 (56)	0.406
	Former smoker	37 (27)	28 (34)	65 (30)	
	Current smoker	22 (16)	9 (11)	31 (14)	
Symptomatic presentation	Macroscopic haematuria	66 (43)	0 (0)	66 (27)	
	LUTS	15 (10)	0 (0)	15 (6)	
	Umbilical infection	31 (20)	0 (0)	31 (13)	
	Umbilical mass	3 (2)	0 (0)	3 (1)	
	Abdominal pain	38 (25)	0 (0)	38 (16)	
	Incidental	0 (0)	92 (100)	92 (37)	
Cystoscopy	Yes	104 (68)	23 (25)	127 (52)	<0.001
Cystoscopy findings	Normal	49 (46)	14 (61)	63 (50)	
	BPH, stone	17 (16)	4 (17)	21 (17)	
	Inflammation	14 (14)	3 (13)	17 (13)	
	Urachal cyst, diverticulum	14 (14)	2 (9)	16 (12)	
	Exophytic tumour	10 (10)	0 (0)	10 (8)	
Imaging method used	CT urography	63 (41)	19 (21)	82 (33)	
	CT with contrast	39 (26)	51 (55)	90 (37)	
	CT without contrast	17 (11)	12 (13)	29 (12)	
	Others^b^	34 (22)	10 (11)	44 (18)	
Type of urachal anomaly^c^	Patent urachus	2 (1)	0 (0)	2 (1)	
	Urachal cyst	25 (16)	13 (14)	38 (15)	
	Urachal sinus	7 (5)	0 (0)	7 (3)	
	Urachal diverticulum	24 (16)	20 (22)	44 (18)	
	Thickened bladder wall	11 (7)	3 (3)	14 (6)	
	Unspecified	84 (55)	56 (61)	140 (57)	
Contrast enhancement^d^	Yes	26 (25)	6 (9)	32 (19)	
Cancer		14 (9)	0 (0)	14 (6)	0.001

*Note*: Figures represent number of patients (% of the column) if not otherwise indicated.

Abbreviations: CT, computed tomography; IQR, interquartile range; LUTS, lower urinary tract symptoms.

^a^ 28 missing.

^b^ Others include magnetic resonance imaging (MRI) or ultrasonography.

^c^ Radiologically.

^d^ Of those who underwent CT urography or with contrast.

Among symptomatic patients, macroscopic haematuria was the most common presenting symptom (43%), followed by abdominal pain (25%) and umbilical infection (20%). Cystoscopy was performed significantly more frequently in symptomatic patients compared with those with incidental findings (68% vs. 25%, *p* < 0.001). The imaging modalities used differed between groups, with CT urography being more frequently used in symptomatic patients (41% vs. 21%), whereas contrast‐enhanced CT was the most frequently used modality in incidental cases (55% vs. 26%). Malignancy was detected exclusively among symptomatic patients (9% vs. 0%, *p* = 0.001).

Macroscopic haematuria showed a strong association with malignancy. Among patients with haematuria, 12 of 66 (18%) were diagnosed with malignancy compared with two of 179 patients without haematuria (1%) (*p* < 0.001). Urachal diverticulum was the most common among the radiologically specified anomalies (18%), followed by urachal cysts (15%), although the majority of lesions were reported as unspecified (57%).

Among the 14 patients diagnosed with malignancy (Table 2), the median age was 65 years (IQR 56–75), and the majority were male (93%). Macroscopic haematuria was present in 86% of cases, while lower urinary tract symptoms (LUTS) accounted for the remaining presentations (14%). On cystoscopy, an exophytic bladder tumour was identified in most patients (71%). Contrast enhancement was present in 36% of malignant lesions. Surgical treatment included TURBT in 44% of patients, cystectomy in 28% and biopsy alone in 28%. The most frequently reported radiological finding among patients with bladder malignancy/PUNLMP was bladder wall thickening (5/14, 36%), followed by urachal diverticulum (3/14, 21%) and urachal cyst (1/14, 7%); the remaining 5/14 (36%) were unspecified. Papillary urothelial carcinoma was the most common histological subtype (50%), followed by squamous cell carcinoma (22%). More than half of tumours were muscle‐invasive or more advanced at diagnosis (≥T2 disease; 57%).

**TABLE 2:** Descriptive parameters of all patients with urological malignancy (n = 14) during follow‐up period.

Variable		Overall
Age (years)	Median (IQR)	65 (56‐75)
Gender	Female	1 (7)
Follow‐up (years)	Median (IQR)	9 (6 ‐13)
Death during follow‐up	Yes	7 (50)
Smoking status^a^	Never smoked	7 (58)
	Former smoker	3 (25)
	Current smoker	2 (17)
Symptomatic presentation	Macroscopic haematuria	12 (86)
	LUTS	2 (14)
Cystoscopy finding	Exophytic tumor	10 (71)
	Urachal diverticulum	3 (21)
	Non‐specific	1 (7)
Contrast enhancement	yes	5 (36)
Radiological finding	Urachal diverticulum	3 (21)
	Urachal cyst	1 (7)
	Thickened bladder wall	5 (36)
	Unspecified	5 (36)
Surgical procedures	Biopsy	4 (28)
	Cystectomy	4 (28)
	TURBT	6 (44)
Type of cancer	Squamous cell carcinoma	3 (22)
	Mucinous adenocarcinoma	1 (7)
	Papillary urothelial cancer	7 (50)
	Small cell neuroendocrine carcinoma	1 (7)
	Low malignant potential (LMP)	2 (14)
TNM	LMP + TaG1‐2	6 (43)
	T2+	8 (57)

*Note*: Figures represent number of patients (% of the column) if not otherwise indicated.

Abbreviations: IQR, interquartile range; LUTS, lower urinary tract syndrome; TNM, tumour node metastasis; TURBT, transurethral resection of bladder tumour.

^a^ Two missing cases.

In univariate Firth penalized logistic regression analysis (Table 3), symptomatic presentation (OR 19.2, 95% CI 2.52–2468.4, *p* = 0.001), male sex (OR 4.34, 95% CI 1.03–40.2, *p* = 0.045), contrast enhancement (OR 4.07, 95% CI 1.24–12.3, *p* = 0.022) and macroscopic haematuria (OR 16.3, 95% CI 4.69–85.1, *p* < 0.001) were significantly associated with malignancy. Age and smoking status were not significantly associated with cancer risk. In the exploratory multivariable model, macroscopic haematuria was the only variable that retained a statistically significant association with bladder malignancy/PUNLMP (OR 5.29, 95% CI 1.10–39.5, *p* = 0.036), albeit with a wide confidence interval. The associations for symptomatic presentation, sex, smoking history and contrast enhancement were attenuated and no longer statistically significant after adjustment.

**TABLE 3:** Univariate and multivariable Firth penalized logistic regression analysis of predictors of malignancy in urachal anomalies.

Variable	Univariate OR (95% CI)	*p* value	Multivariable OR (95% CI)	*p* value
Symptomatic presentation (vs incidental)	19.2 (2.52–2468.4)	0.001	5.98 (0.42–861.2)	0.202
Age (per year)	1.02 (0.99–1.05)	0.122	0.99 (0.95–1.04)	0.737
Male sex (vs. female)	4.34 (1.03–40.2)	0.045	3.20 (0.62–32.7)	0.177
Smoking (ever vs. never)	0.92 (0.28–2.84)	0.882	0.72 (0.20–2.41)	0.595
Contrast enhancement (yes vs. no)	4.07 (1.24–12.3)	0.022	2.90 (0.72–10.8)	0.128
Macroscopic haematuria (yes vs. no)	16.3 (4.69–85.1)	<0.001	5.29 (1.10–39.5)	0.036

Figure 3a demonstrates that urachal anomalies were diagnosed across a broad age range, with most cases occurring in middle‐aged and older adults. Median age at diagnosis did not differ significantly between groups; however, Figure 3b suggests an apparent difference in the shape of their age distributions.

**FIGURE 3:**
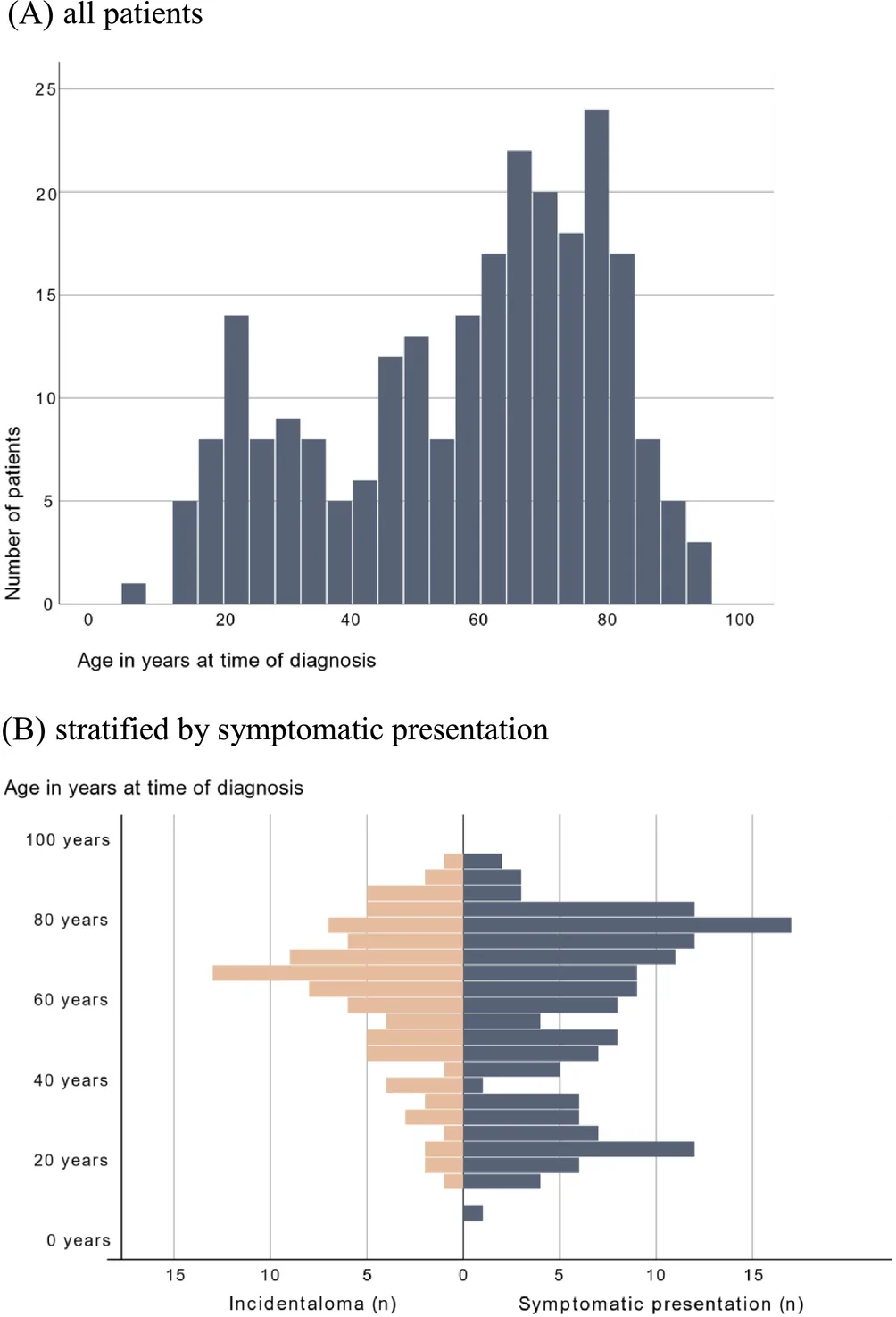
Distribution of age at time of diagnosis of urachal anomaly.

## 4 DISCUSSION

In this cohort of 245 patients with urachal anomalies, most patients were symptomatic, while a smaller proportion were incidental findings. Malignancy occurred only among symptomatic individuals, and the most common symptom at presentation was macroscopic haematuria. In an exploratory adjusted multivariable Firth regression analysis, macroscopic haematuria remained the only variable independently associated with malignancy.

Across the available literature, reported detection rates of urachal anomalies vary widely, depending on study design and population.^2^, ^7^, ^11^, ^16^, ^17^ Retrospective ultrasonography‐based studies in paediatric populations suggest a birth prevalence of approximately 1%–1.5%.^11^, ^17^ Although spontaneous regression has been documented,^17^ the extent to which lesions persist into adulthood is unknown. Stopak et al. reported a marked increase in diagnosed urachal anomalies in children over a 10‐year period, attributing this primarily to increased clinical awareness and improvements in ultrasonography rather than a true rise in prevalence.^2^ Our cohort represents radiologically detected urachal anomalies rather than true prevalence, as asymptomatic lesions remain undetected without imaging. Several malignancies were diagnosed at the index examination, and our findings should therefore be interpreted as the malignancy yield associated with radiological detection of urachal anomalies rather than the risk of malignant transformation over time.

### 4.1 Absence of malignancy in incidental cases

The most important finding in the present study is that malignancy occurred exclusively among symptomatic patients and that macroscopic haematuria was the only independent predictor in the adjusted model. This underscores the central clinical importance of macroscopic haematuria as a marker of underlying malignancy. Although the observed odds ratio was high, confidence intervals were wide because of the limited number of malignant cases. Consequently, the regression findings should be interpreted cautiously. Only 14 malignant/PUNLMP events were observed relative to the number of candidate predictors, increasing the risk of overfitting. Furthermore, symptomatic presentation and macroscopic haematuria are conceptually related, since haematuria formed part of the definition of symptomatic disease. Therefore, the regression analysis should primarily be viewed as supporting the descriptive observation that haematuria is clinically important rather than as a robust prediction model. A retrospective single‐centre study of 45 adults reported that 67% of urachal anomalies were incidental of whom most were managed conservatively.^6^ Malignancy was identified in three patients, and all non‐operatively managed lesions remained stable during follow‐up. In a large paediatric cohort of 721 patients reported by Gleason et al.,^11^ 667 (92%) urachal anomalies were detected incidentally using ultrasound, of whom 61 (8.3%) underwent surgical excision. They estimated that 5721 excisions would be needed to prevent one case of cancer. Evidence from these two studies broadly aligns with our findings, suggesting a low risk of malignant transformation of urachal anomalies and supporting a risk‐stratified, conservative management approach.^6^, ^11^ Ashley et al. evaluated 176 patients with urachal anomalies, of whom 130 were adults, and reported malignancy in 51% of adult cases, with macroscopic haematuria and age >55 years as independent predictors.^3^ This markedly higher rate likely reflects referral bias, as the cohort was drawn from a major tertiary referral centre, and detection bias due to limited use of cross‐sectional imaging over the long study period (1951–2005). Both these biases would have reduced detection of benign incidental anomalies relative to symptomatic, advanced cases. Nevertheless, this study identifies haematuria as an important clinical indicator of underlying malignancy. Fode et al. reported a notable complication rate following robot‐assisted laparoscopic resection in symptomatic patients in a small case series, with malignancy identified in only one of nine cases.^18^ This finding further supports an individualized rather than routine surgical approach to symptomatic lesions.

### 4.2 Non‐urachal histology predominates despite limited impact of risk factors

Male sex, increasing age, smoking and occupational carcinogens are well‐established risk factors for bladder cancer.^19^, ^20^ In our cohort, however, age, sex and smoking status were not significantly associated with malignancy after adjustment. Given the limited number of malignant events, this should not be interpreted as evidence against such associations, as the study was likely underpowered to detect them.

In our cohort, most malignancies were urothelial rather than urachal adenocarcinomas. This contrasts with the urachal carcinoma literature, where adenocarcinoma predominates.^21^ This pattern suggests that the detected cancers are likely to reflect the baseline risk of bladder cancer within the studied population, rather than direct malignant transformation originating from the urachal remnant itself. In other words, urachal anomalies may often represent an incidental coexisting finding rather than a premalignant lesion.

This has direct implications for clinical management, particularly in light of the ongoing debate regarding prophylactic excision of asymptomatic urachal remnants.^3^ If malignancy rarely originates from the urachus itself, the rationale for routine surgical removal as a preventive measure becomes less compelling. Instead, the observed association between urachal anomalies and malignancy may be explained by shared risk factors or by increased diagnostic scrutiny in symptomatic patients.

Macroscopic haematuria emerged as the key clinical predictor of malignancy which is consistent with previous haematuria literature,^22^, ^23^ underscoring the need for thorough evaluation. In contrast, imaging features such as contrast enhancement lacked independent predictive value, highlighting their limited specificity. These findings support therefore a more individualized approach, where surgical intervention is guided by clinical presentation and suspicion of malignancy rather than the mere presence of an urachal anomaly.

Bladder adenocarcinoma is rare, accounting for approximately 0.5–2% of bladder cancers,^9^, ^24^ of which a substantial proportion are of urachal origin. A previously published institutional cohort identified four adenocarcinomas among 785 bladder cancer cases (0.5%),^25^ of which one overlapped with the present cohort, a proportion broadly consistent with the expected urachal share. The remaining three were located outside the expected location of the urachus and were not described as urachal in either medical records or radiological reports.

These findings support a conservative, risk‐adapted management strategy. Patients with macroscopic haematuria are at increased risk of malignancy and require thorough evaluation, including cystoscopy, with a low threshold for surgical intervention. In contrast, asymptomatic patients with incidental findings and no suspicious imaging features appear to have a very low risk and may be safely managed with observation. Patients with non‐specific symptoms or equivocal imaging represent an intermediate group requiring individualized assessment and follow‐up.

This study is strengthened by its comprehensive clinical data which enabled detailed assessment of presenting symptoms, diagnostic pathways and outcomes. The use of Firth penalized logistic regression is another methodological strength, as it is well suited for analyses involving rare events and small sample sizes, thereby reducing bias in the presence of rare events and small sample sizes.

Several limitations should also be acknowledged. The low number of malignancy cases reduced statistical power and resulted in wide confidence intervals for some estimates. In addition, the retrospective design introduces potential selection bias and a risk of incomplete data collection. Diagnostic work‐up and imaging practices changed over time, which may have influenced both detection rates and classification of urachal anomalies.

A further limitation is differential verification bias, as symptomatic patients were substantially more likely to undergo cystoscopy than patients with incidental or asymptomatic findings. This may have increased malignancy detection in symptomatic patients while potentially underestimating malignancy among conservatively managed individuals without cystoscopic evaluation. The radiological subtyping of lesions was based on original reports rather than structured re‐review of the images and was thus limited by the quality and terminology of the historical reports. Most lesions were categorized as unspecified due to insufficiently detailed descriptions, limiting possible conclusions regarding subtype‐specific malignancy risk. Finally, international variation in the incidence of urachal adenocarcinoma has been reported,^26^ which may limit the generalizability of these findings to other populations and healthcare contexts.

## 5 CONCLUSION

No malignancies were observed among adults with incidentally detected/asymptomatic urachal anomalies during the study period. Malignancies in the total cohort occurred only in symptomatic patients, predominantly those with macroscopic haematuria, and were predominantly of non‐urachal histologies. These findings support a risk‐adapted approach after appropriate initial evaluation, but they do not prove that the long‐term transformation risk is zero.

## AUTHOR CONTRIBUTIONS

*Conception and design:* Suleiman Abuhasanein. *Acquisition of data:* Per Blomqvist, Lala Joulhay Ian, Emelie Holmin. *Analysis and interpretation of data:* Suleiman Abuhasanein, Per Blomqvist. *Drafting of the manuscript:* Suleiman Abuhasanein, Per Blomqvist. *Critical revision of the manuscript for important intellectual content:* Suleiman Abuhasanein, Per Blomqvist, Lala Joulhay Ian, Emelie Holmin. *Obtaining funding and supervision:* Suleiman Abuhasanein.

## CONFLICT OF INTEREST STATEMENT

The authors have no conflict of interest to declare.

## ETHICS STATEMENT

All authors declared that the research was conducted according to the principles of the World Medical Association Declaration of Helsinki ‘Ethical Principles for Medical Research Involving Human Subjects’. The study was approved by the Swedish Ethical Review Authority (File No. 2025‐00079‐01).

## Data Availability

The datasets used and/or analysed during the current study are available from the corresponding author on reasonable request.
