# Correction to “Malignancy yield and clinical predictors in radiologically detected adult urachal anomalies: A retrospective cohort study”

**DOI:** 10.1002/bco2.70302

**Published:** 2026-10-26

Blomqvist P , Joulha Ian L , Holmin E , Abuhasanein S . Malignancy yield and clinical predictors in radiologically detected adult urachal anomalies: A retrospective cohort study. BJUI Compass. 2026; 7(10):e70284. 10.1002/bco2.70284 PMC1363953142840620

In the article cited above, one of the authors' surnames has been published incorrectly. The author is currently listed as **Lala Joulhay Ian**, whereas the correct name is **Lala Joulha Ian**.

The online version of this article has been corrected accordingly.

We apologize for this error.

